# Structural and Functional Thalamic Changes in Progressive Supranuclear Palsy

**DOI:** 10.1192/bjo.2022.252

**Published:** 2022-06-20

**Authors:** Sean YW Tan, P Simon Jones, David J Whiteside, James Rowe, Timothy Rittman

**Affiliations:** 1 Cambridge University Hospitals NHS Trust, Addenbrooke's Hospital, Cambridge, United Kingdom; 2 Department of Clinical Neurosciences, University of Cambridge, Cambridge, United Kingdom; 3 MRC Cognition and Brain Sciences Unit, University of Cambridge, Cambridge, United Kingdom

## Abstract

**Aims:**

Studies of thalamic structure and function in Progressive Supranuclear Palsy (PSP) suggest it may play a role in key aspects of the clinical syndrome. This study examined thalamic changes across PSP phenotypes investigating (i) thalamic atrophy (ii) thalamic functional connectivity and (iii) the relationship between thalamic structural and functional connectivity changes with clinical severity.

**Methods:**

**Participants**

92 participants with PSP [63 PSP-Richardson's Syndrome (RS), 24 PSP-cortical, 5 PSP-subcortical] and 104 age-matched controls were recruited from the Cambridge Centre for Parkinson's Plus Disorders cohort. Clinical assessments and imaging were conducted within 1 year of diagnosis.

**Structural Analysis**

Thalamic volumes (TVs) were obtained using FreeSurfer. Bayesian multiple regression (brms, R) was used to model (i) mean TVs (ii) group differences in mean TVs (iii) relationships between Z-standardised clinical scores and TVs with age, gender, and total grey matter as covariates.

**Functional Analysis**

Voxel-wise seed-based functional connectivity of the thalamus used the Functional Magnetic Resonance Imaging Expert Analysis Tool (FEAT) in FMRIB's Software Library (FSL). Inter-group differences and relationships between clinical scores and functional connectivity for each group were assessed using a general linear model with age and gender as covariates.

**Results:**

**Structural Analysis**

TVs for all PSP subgroups were smaller than controls. No differences between PSP subgroups were detected. There was evidence for a relationship between TVs for the entire PSP group and Revised Addenbrooke's Cognitive Examination (ACER) scores [ß = 0.28, 95% credible interval (CI) = 0.04–0.53]. Subgroup analysis showed evidence for a relationship between ACER scores and TVs in PSP-RS [ß = 0.33, 95% CI = 0.09–0.57] and PSP-cortical [ß = 0.46, 95% CI = 0.12–0.83] phenotypes. A negative influence of TVs on total PSP rating scale scores was found for the PSP cohort a whole [ß = −0.51, 95% CI = −1.00 – −0.02].

**Functional Analysis**

PSP patients as a group showed decreased thalamic functional connectivity in higher cortical regions. Subgroup analysis revealed decreased connectivity in those areas compared to controls but in distinct distributions and magnitude. Increased thalamic connectivity with the middle temporal gyrus correlated with ACER scores for PSP patients as a group and in the PSP-cortical subtype.

**Conclusion:**

Thalamic volume loss is a prominent aspect of PSP and is associated with a wide network of changes in functional connectivity that may be distinct between PSP subtypes. Changes in thalamic structure and function predict clinical severity, particularly in PSP-RS and PSP-cortical subtypes.

